# Medication adherence influencing factors—an (updated) overview of systematic reviews

**DOI:** 10.1186/s13643-019-1014-8

**Published:** 2019-05-10

**Authors:** Alina Gast, Tim Mathes

**Affiliations:** 1 Institute for Research in Operative Medicine (Witten/Herdecke University), Ostmerheimer Str. 200, 51109 Cologne, Germany; 2 Institute for Health Economics and Clinical Epidemiology of the University of Cologne, Gleueler Str. 176-178, 50935 Cologne, Germany

**Keywords:** Adherence, Compliance, Overview, Systematic review, Oral medication, Influencing factors, Physical chronic conditions

## Abstract

**Background:**

Non-adherence negatively affects the efficacy, safety and costs of therapies. Non-adherence is a multifactorial problem. This systematic review (SR) of SRs (overview) aims to identify factors that can influence the adherence of adult patients with chronic physical diseases.

**Methods:**

We performed a systematic literature search in MEDLINE and Embase on June 13, 2018. We included SRs on the factors that can influence adherence in adult patients taking oral medications for treating physical chronic diseases. Two reviewers independently selected studies according to pre-defined inclusion criteria. Two reviewers independently assessed the risk of bias with the ROBIS tool. Data were extracted in standardized tables previously piloted by one reviewer and verified by a second reviewer. We synthesized data in tables in a structured narrative manner.

**Results:**

We included 21 SRs on eight different conditions. We rated eight SRs to be at low risk of bias and 13 to be at high risk of bias. Although higher education, employment, higher financial status and marriage/partnership mostly showed a positive effect on adherence, the impact was unclear because of the high uncertainty of the underlying evidence. The evidence indicates that socioeconomic status and social support might have a positive impact on adherence and that belonging to an ethnic minority might have a negative impact on adherence. Therapy-related factors (e.g., intake regime) and disease-related factors (e.g., duration) mostly showed no impact on adherence. Analysis of gender showed inconsistent results. Age might have a concave relation to adherence, i.e., adherence is lowest in very young and very old people. Depression has a negative impact on adherence. Impacts of other mental and physical comorbidities were uncertain. Co-payments (any or higher) have a negative impact on adherence. In contrast, the impacts of medication costs and insurance status were uncertain.

**Conclusion:**

This overview analyses factors that might impact adherence to oral therapies in adult patients with physical chronic diseases. Our overview suggests that there is a social gradient in adherence. However, for most factors, the evidence was not conclusive due to the risk of bias, inconsistency or imprecision.

**Electronic supplementary material:**

The online version of this article (10.1186/s13643-019-1014-8) contains supplementary material, which is available to authorized users.

## Background

Medication adherence can be defined as the extent to which a patient’s behaviour corresponds with the prescribed medication dosing regime, including time, dosing and interval of medication intake [1, 2]. Non-adherence is a crucial point for the success and safety of many therapies [3–5]. Medication non-adherence is a widespread problem that causes high costs worldwide [5–10]. Especially in chronic conditions with long-term therapies, adherence is important to achieve target outcomes but is often low [10].

Adherence is a multifactorial phenomenon that can be influenced by various factors. These factors can be divided into five different dimensions: social and economic factors, therapy-related factors, disease-related factors, patient-related factors and health care system-related factors [10, 11]. Some factors can have an influence on intentional non-adherence (conscious decision not to take the medication; e.g., because of high co-payments), while others can have an influence on non-intentional (forgetting) non-adherence (e.g., forgetfulness because of mental comorbidity).

Insights into the factors that might have a negative influence on adherence are important for several reasons. First, this information can support the identification of patients at high risk for non-adherence. Second, it can support the identification of possible adherence barriers that might be eliminated. Third, it can support the development of individually tailored adherence-enhancing interventions.

The objective of this (updated) overview (systematic review [SR] of systematic reviews) was to identify those factors that influence adherence to oral drugs in patients with physical chronic diseases. Given the considerable amount of literature in this field, this updated overview provides a current and compact overall view on this topic.

## Methods

There was no published protocol for this overview. Unless otherwise indicated, all described methods were specified before conducting the overview. This overview was not registered.

### Information sources

This overview is a focused updated version of an overview published by our research team in 2014 [12]. This overview is reported according to the Preferred Reporting Items for Overviews of systematic reviews (OoSRs), including the harms checklist [13].

We performed a systematic literature search in MEDLINE (via PubMed) and Embase (via Embase). The complete search strategy, including the applied search limits, is provided in Additional file 1. In contrast to our previous search filter, we included unspecific terms for influencing factors (e.g., factors, predictors) as well as specific terms (e.g., gender, age) because we focused only on certain pre-defined influencing factors (for the reasoning, see the “Study Selection” section). We anticipated that these parameters would lead to a higher sensitivity compared with the search for the previous overview version. In addition, the search was performed without limiting the publication date. We performed the search of the electronic databases on June 13, 2018. In addition to the electronic searches, we crosschecked the references of all included SRs.

### Study selection

We selected SRs according to the following predefined inclusion criteria:

1. Patients: Adult patients (≥ 16 years) with physical chronic diseases. We considered every physical chronic illness. We excluded SRs that analysed children (if > 20% of the included studies analysed children), and considered only patients with acute conditions or considered only patients with mental illnesses.
2. Medication: Oral drug intake (at least 50% of patient population)
3. Exposure: Pre-specified (see the text below) potential influencing factors for adherence. We defined a factor as any exposure that is not controlled by the study investigator
4. Outcome: Implementation adherence (correct dose, timing and/or frequency of intake) [2]
5. Study type: SRs (definition: systematic literature search in at least one electronic database and assessment and documentation of risk of bias of included studies) of quantitative studies
6. Publication language: English or German

We aimed to summarize the evidence for factors that are widely applicable across different conditions, therapies and regions/settings. Therefore, we limited our overview to unrelated factors of therapy and disease, i.e., we excluded factors that likely strongly vary depending on disease (e.g., symptoms), therapy (e.g., side effects) or health care system (e.g., insurance type). Compared with the previous version, we narrowed the scope by considering only factors for which there were some indices for an influence in the previous broad overview [12]. We chose the following factors: age, gender, ethnic status, education, employment, financial status/income, marital status/not living alone, social support, measure of intake complexity (e.g., number of tablets, number of medications, frequency of intake), duration of therapy, duration of disease, comorbidity, co-payments, medication costs and insurance status (insured/not insured).

In addition to these pre-defined eligibility criteria, a further criterion was defined post hoc during study selection. Both reviewers agreed to exclude those SRs that reported only the number of statistically significant studies (e.g., 10 studies showed a statistically significant effect of gender) without reporting effect sizes and the total number of studies on a certain comparison (e.g., 12 studies analysed gender). The decision to exclude studies that were reported in this way was made because the results could have been highly biased by selective reporting otherwise.

The study selection (title/abstract screening and full-text screening) was performed by two reviewers independently. Any differences between the reviewers were discussed until consensus.

### Data collection

All data were extracted using standardized extraction forms piloted beforehand. Data were extracted by one reviewer, and completeness and accuracy were verified by a second reviewer. Any disagreements were discussed until consensus. For each SR, we extracted the following characteristics: condition/medication, eligibility criteria for primary studies (only other than our applied inclusion criteria), search period and any search limits.

The results were extracted according to the type of evidence synthesis. For all meta-analyses, we extracted pooled effect estimates with 95% confidence intervals, tests and measures for statistical heterogeneity, the number of included studies and the number of patients included in the meta-analyses. In the case that the included SR performed only a narrative synthesis, we used modified vote counting to extract the results. This method has been suggested for presenting results of quantitative synthesis and overcoming problems of simple vote counting [14, 15]. We extracted information on the effect direction, total number of included primary studies showing a certain effect direction, statistical significance of primary studies (*p* < 0.05) showing the effect direction and total number of primary studies that analysed a certain factor.

All data in the tables were harmonized so that the influence on adherence (not non-adherence) refers to an increase in the factor regardless of whether the factor is positive (e.g., socioeconomic status) or negative (e.g., co-payments).

### Risk of bias assessment of individual studies and across studies

We used the Risk of Bias in Systematic Reviews (ROBIS) tool to assess the included SRs [16]. The ROBIS tool is based on three phases. Phase 1 aims to assess the relevance of the SR. For this purpose, the relevance of the research question should be assessed. This optional phase was skipped in this overview because the relevance was already completely covered by the eligibility criteria. Phase 2 comprises four different domains (domain 1: study eligibility criteria, domain 2: identification and selection of studies, domain 3: data collection and study appraisal, and domain 4: synthesis and findings) and aims to identify biased areas in the SRs. In the final phase 3, the assessor judges whether the whole SRs is at risk of bias. In addition to the results of phase 2, three additional signalling questions should be considered in phase 3. These three signalling questions refer to the discussion/interpretation of the SRs. We did not extract any data from the discussion/interpretation; therefore, we did not consider these signalling questions in the overall judgement. Thus, the overall judgement of risk of bias is exclusively based on the results of phase 2 [17]. The ROBIS tool was applied by two independent reviewers (TM, AG). Disagreements were resolved by discussion. TM was also an author of two of the included SRs. To ensure an objective assessment, the risk of bias assessment of these SRs was performed by a reviewer other than TM.

### Synthesis of results

For all factors, a summary evaluation of the influence on adherence across SRs was made. The evidence for an impact was rated by considering the following criteria that were inspired by the GRADE [18] criteria.

- Risk of bias of the included SRs and their included primary studies. In primary studies, we considered in particular adjustment for confounding, missing data and adherence measurements
- Imprecision (statistical certainty, amount of information on a certain factor [number of primary studies and SRs, effect size)])
- Inconsistency (within and between SRs, e.g., due to different adherence measures)

Based on these criteria, the effects were rated as *robust evidence for an impact*, *some evidence for an impact*, *probably no impact* or *uncertain impact*. The impact rating was performed by two reviewers.

Overlaps (multiple included primary studies) were assessed by creating a cross table of all included SRs and their primary studies. In addition, the corrected covered area (CCA) was calculated. The CCA is a value that indicates the proportion of overlapping primary studies. It is calculated as follows: $\mathrm{CCA}=\frac{\left(N-r\right)}{\left(r\times c-r\right)}$; *N* = number of primary studies (includes multiple counting); *r* = number of index studies (defined as first-time primary study); and *c* = number of included systematic reviews. The CCA can assume a value between 0 and 100%. The smaller the value is, the lower the overlap. Conversely, the higher the value is, the greater the overlap [19].

## Results

### Study selection

**Fig. 1:**
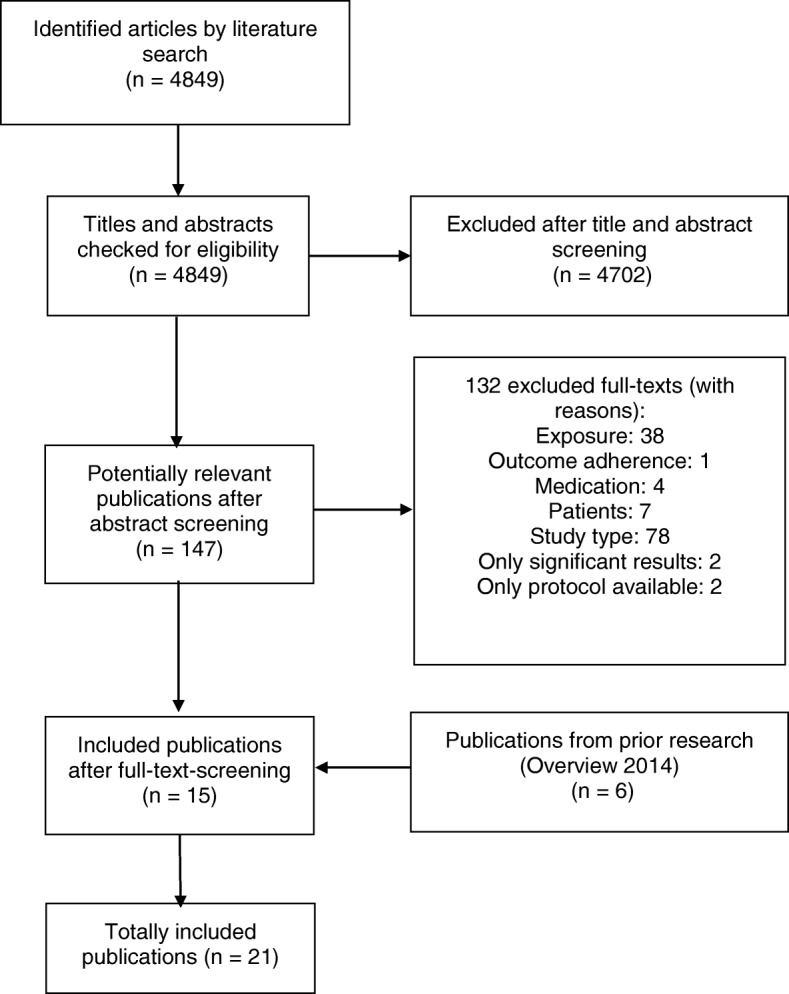
Flowchart of the study selection

The electronic literature research resulted in 4849 hits after removal of duplicates (including hits from the previous search). After title and abstract screening, 4702 articles were excluded, and 147 were judged to be potentially relevant. The full texts of these articles were screened in detail. Fifteen SRs met all eligibility criteria and were included in this overview. Most SRs were excluded because a methodological quality assessment of the included primary studies was not performed or factors other than our pre-specified influencing factors were investigated. In addition to the 15 newly identified relevant SRs, six SR of the previous overview were included. Finally, 21 SRs were included in this overview [20–40]. The process of study selection is illustrated in the PRISMA flowchart [41] (Fig. 1).

### Characteristics of the included systematic reviews

The following conditions and medications were considered: chronic non-malignant pain [35], cardiovascular diseases (e.g., coronary artery disease, hypertension, diabetes mellitus) [21–26, 29, 30, 33, 37], Parkinson disease [36], hepatitis C [27], oral anticancer agents [28, 39], inflammatory arthritis [38], HIV/AIDS [31, 32, 34] and chronic diseases [20]. Sinnott et al. did not restrict the condition or medication but included all studies on publicly insured patients who were exposed to co-payments for medications [40]. Of the 21 included SRs, 14 only synthesized the results narratively, and seven performed a meta-analysis. The characteristics of all included SRs are presented in Table 1. A list of excluded studies is available in Additional file 2.

**Table 1:** Study characteristics

Study	Search period	Inclusion criteria (patients and medication marked in italics)
Explorative systematic reviews		
Aziz 2016	Not limited to February 2015	*Patients with chronic conditions and different payment schemes*
		*Human adults*
		Published in English
		Evaluation of the effect of medication cost or method of payment on medication adherence
		Clear description of study population and methodological approach
		Only studies without a adherence intervention
		Only randomized controlled trials, cross-sectional, longitudinal and observational/prospective or retrospective cohort studies
		Only original research (review articles, thesis, commentaries, editorial letters, and case studies were excluded)
Broekmans 2008	Not limited to December 2006	*Adult patients with chronic non-malignant pain*
		*Adult patients with prescribed pain medication*
		Original research
Chen 2015	January 1990 to September 2013	*Patients after acute coronary syndrome getting secondary prevention pharmacotherapy*
		Humans aged ≥ 18 years
		Subjects hospitalized for an acute coronary syndrome
		Prescription of at least one specified evidence-based medication after hospital discharge (beta-blocker, lipid-lowering agents, antiplatelet agents, ACEIs or ARBS)
		Report of medication usage after hospital discharge
		Measuring medication adherence and reporting its method of measurement
		Only secondary adherence/non-adherence (not initiation)
		At least 2 months follow-up
		Specific follow-up time for calculating medication adherence
		Calculation of medication adherence of patients with at least one filled prescription for the medication of interest during the follow-up time
		All study designs
		Only original research
		Only analysis of the original study population
		Publication in a peer-reviewed journal
Daley 2012	Not limited to January 2012	*Patients with Parkinson* *All ranges and duration of anti-parkinsonian treatments*
		All age ranges
		Published in English
		Presenting quantitative/qualitative data
Gourzoulidis 2017	Not limited to NR	*Patients with diabetes mellitus or heart failure*
		Different study types including retrospective, longitudinal observational cohort or cross-sectional studies (no reviews, meta-analyses, editorials, comments or letters to the editor)
		Co-payment-interventions (introduction of co-payments or increases/decreases in existing co-payments)
		Studies assessing the impact of co-payments on adherence
		Exclusion of other types of cost-sharing, co-insurance, deductibles or caps
		Exclusion of economic evaluations and treatment interventions
		Only English and full-text published articles
Jaam 2017	Not limited to May 2016	*Adults patients* (≥ 18 years old*) with diabetes mellitus ty*pe *1 or 2 living in the Middle East and North African region*
		Only original research reporting qualitative or quantitative data
		Studies investigating factors associated with medication adherence
		Patients receiving anti-diabetic medication
Krueger 2015	Not limited to March 2014	*Adult patients with chronic heart failure*
		Studies analysing the relationship between age and medication adherence
		Studies relating to pharmacological adherence
		Only original research
		Poor quality studies were excluded
		Published in every language
Maimaris 2013	Not limited to May 2013	*Adult population (general or on hypertension treatment)*
		Studies reporting on effects of national or regional (not individual or organisational levels) health system level arrangements (interventions, policies, or programs) on *hypertension control*
		Adult population, including general population, population on treatment and population with specific comorbidities
		Quantitative studies
		Quantitative studies must report a measure of association between health system arrangement and at least one hypertension outcome of interest
		Different study types including controlled trials, cohort studies and cross-sectional studies
		Published in every language
Mann 2014	Not limited to March 2013	*Adult patients with cardiovascular-related chronic conditions* (coronary artery disease, hypertension, diabetes, hypercholesterolemia, cerebrovascular disease)
		Studies assessing drug insurance (intervention) against a comparator group (including various cost-sharing strategies like co-payments, fixed co-payments, co-insurance, deductibles, caps, coverage gaps)
		Different study designs including randomized controlled trials, non-randomized controlled trials, before-after-studies, interrupted time series
		Studies reporting on medication adherence, clinical outcomes, quality of life, health care utilization or costs
		Studies not focussing on health policy, value-based insurance or reference based pricing English published
Mathes 2014(a)	Not limited to December 2012	*Hepatitis C-infected patients*
		Adult patients with hepatitis C
		Patients getting medication regimes containing ribavirin
		Every study type with quantitative measure of patient implementation adherence Studies analysing potential adherence influencing factor/s
		Studies conducted in WHO-mortality Stratum A (very low child mortality and low adult mortality)
		Published in English or German
Mathes 2014(b)	Not limited to December 2012	*Patients taking oral anticancer agents*
		Patients ≥ 18 years old with malignant neoplasms
		Patients taking oral anticancer agents
		Studies analysing potential adherence influencing factor/s
		Every study type with quantitative patient adherence measure (no interventional trials)
		Studies not exclusively referring to intentional non-adherence measures
		Published in English or German
Oosterom-Calo 2013	Not limited to August 2010	*≥ 50% heart failure patients*
		Quantitative results were reported
		Studies of at least fair quality
		Evaluations of interventions were not the main purpose
		No descriptive study
		No review paper
		Published in English
Pasma 2013	Not limited to February 2011	*Inflammatory arthritis patients* Used a reproducible definition or validated instrument to measure adherence Provided a statistical measure to reflect the strength of the association between the determinant and adherence No letters, editorials, reviews, RCTs, case reports, qualitative studies and opinion articles
Verbrugghe 2012	NR	*Oral anti-cancer drugs*
		Age ≥ 18
		Strong or moderate methodological quality
		Written in English, French, German or Dutch
		Original research articles published between 1990 and April 2012
		Studies not conducted in developing countries
		All study designs
Focused systematic reviews		
Alsabbagh 2014	Not limited to February 2012	*Patients taking antihypertensive drugs*
		Analysis of the influence of socioeconomic status on adherence to antihypertensive medications
		All study designs
		Published in English or French
		Studies used electronic prescription database as source for nonadherence information
		Multivariable modelling
Crawshaw 2016	January 2000 to December 2014	*Adult patients* (> 18 years old) *after acute coronary syndrome* (myocardial infarction and/or unstable angina) *getting secondary prevention pharmacotherapy*
		Cross-sectional, retrospective cohort or prospective cohort studies
		Measure of adherence to cardiac medication (antiplatelet agents, ACE inhibitors, ARBs, beta-blockers, lipid-lowering agents, calcium channel blockers or diuretics)
		Standardised measurement of psychosocial variable
		Assessment of strength of association between psychosocial factors and adherence
		Published in English
Ghidei 2013	NR to July 2012	*Older HIV-infected individuals*
		Only studies with control group
		All study designs excluding case reports
		Only studies with specified cut-off for adherence (≥ 80%)
		Only studies not focussing on psychiatric disorders
		Patient in the older classification aged > 45 years
		Initial use of antiretroviral therapy at or after 1996
		Participations actually on antiretroviral therapy
		Participations without substance abuse
		Peer-reviewed articles Only original research
Hiko 2012	January 1997 to December 2011	*Adults living with HIV/AIDS*
		Adult patients (aged ≥18 years) living with HIV/AIDS
		Patients receiving antiretroviral therapy
		Patients living in developed and developing countries
		Studies identifying determinants of non-compliance regarding antiretroviral therapy (socioeconomic-related, health service-related, psychosocial- and behavioural-related and clinical-related outcome measures)
		Quantitative evidence from observational analytic epidemiological studies (including prospective and retrospective cohort studies, case-control and comparative cross-sectional studies)
		Published in English
Lewey 2013	NR to 04/2010	*Patients receiving statin therapy*
		Studies evaluating adherence to statin therapy and reporting gender, race or ethnicity as a predictor of adherence
		Studies using univariable or multivariable analysis
		Studies reporting quantitative measures of adherence
		Only original data
		Studies reporting adherence to statin therapy and another medication were also included
Nachega 2015	January 1980 to September 2014	Patients receiving antiretroviral therapy
		Every study design
		Patients living with HIV
		Patients receiving antiretroviral therapy
		Studies assessing treatment adherence via objective or self-reporting measures
		Studies considering employment as a possible adherence influencing factor
Sinnott 2013	1946 to September 2012	*Participants received healthcare from a public insurance scheme*
		Comparator group was the same population/similar population who either did not pay co-payments or experienced no increase in co-payment
		The intervention was co-payment; either an increase in an existing co-payment or the introduction of a co-payment (no other types of cost-sharing, for example, co-insurance)
		Studies included were randomised controlled trials, controlled before and after studies, interrupted time series designs, repeated measures designs, and cohort designs

*NR* Not Reported

The 21 SRs included 313 primary studies, and data from these studies were used in this evidence synthesis. The number of index publications was 285 (*r* = 285), which resulted in a primary study overlap estimated by the CCA of approximately 0.5%. The cross table can be found in Additional file 3.

### Risk of bias of the included systematic reviews

**Fig. 2:**
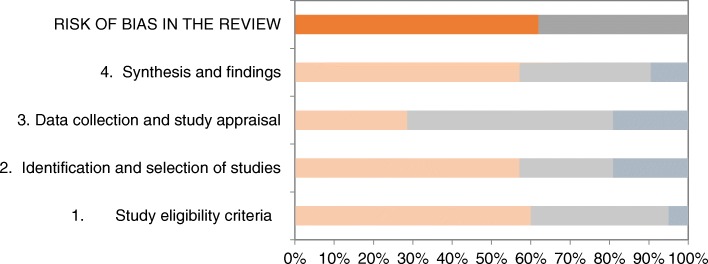
Risk of bias in the systematic reviews. orange: high (risk of bias), grey: low (risk of bias), blue-grey: unclear (risk of bias)

Risk of bias across the SRs was lowest in *domain 3* (*data collection and study appraisal*). In this domain, six SRs were judged to be at high risk of bias. Compared with domain 3, the other domains, including 1 (eligibility criteria), 2 (identification and selection of studies) and 4 (synthesis), were at higher risk of bias across studies. In all these domains, more than 50% of the SRs were at high risk of bias. In particular, imprecise eligibility criteria, inadequate restrictions in the eligibility criteria, inappropriate search strategies, simple vote-counting and no protocols available were the most common reasons for the high risk of bias in these domains. Figure 2 shows the results of the phase 2 ROBIS rating according to the four different domains.

A comparison of the individual SRs shows that only three SRs were at low risk of bias in all four domains [25, 27, 28]. In contrast, 2/3 of all included SRs were at high risk of bias in two or three domains [20, 21, 23, 24, 26, 30, 33, 35, 37–39]. Three SRs were rated to be at high risk of bias in all domains [22, 32, 36]. The results for each included SRs are illustrated in Table 2. We rated the overall risk of bias for eight SRs as low and for 13 SRs as high.

**Table 2:**
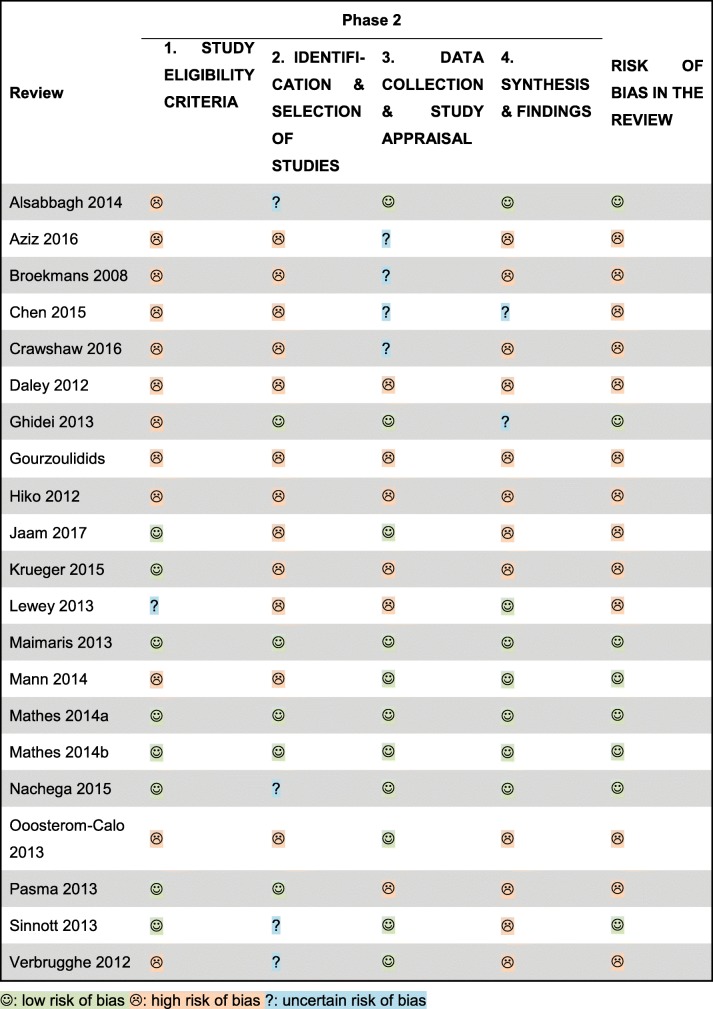
Results of the risk of bias assessment

### Impact of influencing factors of adherence

The evidence synthesis of the analysed factors (according to the different diseases/therapies) is presented in Table 3. The results of each individual included SR are presented in the Additional file 4.

**Table 3:** Evidence synthesis

Factor		Relationship		
		Indication/therapy	Effect direction	Evidence for effect
Social and economic	Education	Parkinson disease	↑	O
		Chronic pain	?	O
		Hepatitis C	↑	O
		HIV	↓	–
		Oral anti-cancer agents	↑	O
			?	O
		Cardiovascular conditions	↑	+
			↓	O
			?	O
	Employed	Hepatitis C	↓	O
		Inflammatory arthritis	?	O
		HIV	↑	+
		Cardiovascular conditions	↑	O
			↓	O
			?	O
	Ethnic status	Hepatitis C	?	O
			Others > African American	O
		Inflammatory arthritis	White > others	+
		HIV	White > Black	+
		Oral anti-cancer agents	White > Black	O
			White > Asian	–
			White > Hispanic	–
			White > non-White	O
			African American > others	O
			Non-White > others	O
		Cardiovascular conditions	White > others	++
			Non-Asian > Asian	O
			Major ethnic groups > ethnic minorities	+
	Financial status/income	Parkinson disease	↑	O
		Hepatitis C	↓	O
		Chronic conditions	↑	+
		Oral anti-cancer agents	↑	+
		Cardiovascular conditions	↑	O
			?	O
	Socioeconomic status	Inflammatory arthritis	↓	O
		Oral anti-cancer agents	?	O
			↑	O
		Cardiovascular conditions	↑	+
	Married/not living alone	Parkinson disease	↑	O
		Inflammatory arthritis	↑	O
		Chronic conditions	?	O
		HIV	↓	O
		Oral anti-cancer agents	↓	O
			↑	O
			?	–
		Cardiovascular conditions	↑	O
			?	O
	Social support	Inflammatory arthritis	↑	O
		Oral anti-cancer agents	?	O
			↑	O
		Cardiovascular conditions	↑	O
			↕	O
Therapy related	Duration of therapy	Oral anti-cancer agents	↓	–
			?	O
			1 year > 3 or 5 years	–
			More than 2 years > 0–2 years	–
	Frequency of intake	Parkinson disease	↑	O
		Inflammatory arthritis	?	O
		Cardiovascular conditions	?	O
	Number of pills taken per day	Cardiovascular conditions	?	O
	Number of tablets	Oral anti-cancer agents	?	O
			2 > 1	O
	Different medications	Parkinson disease	↓	O
		Chronic pain	↓	O
			↑	O
		Inflammatory arthritis	↑	O
		Oral anti-cancer agents	↑	O
			↓	O
			?	O
		Cardiovascular conditions	↓	+
			?	O
	Taking medication at meals	Oral anti-cancer agents	↓	O
Disease related	Duration of disease	Chronic pain	?	O
		Hepatitis C	↑	O
		Inflammatory arthritis	↓	–
		Oral anti-cancer agents	↓	–
			↑	O
			?	–
		Cardiovascular conditions	↑	O
			↓	O
			?	–
Patient related	Age (years)	Parkinson disease	↑	+
		Chronic pain	↑	O
		Hepatitis C	?	O
		Inflammatory arthritis	↑	O
			55–64 > others	O
		Chronic conditions	↑	O
			↓	O
			65 and older > younger than 65	O
		HIV	18–40 < age more than 41	O
			Age less than 45 vs. more than 45	+
		Oral anti-cancer agents	Middle age > very old (≥ 75) > young (≤ 45)	+
			Middle age (41–60) > others	O
			Less than 45 < others	O
			Less than 46 or more than 85 > others	O
			↑	
			↓	O
			?	O
				O
		Cardiovascular conditions	↓	O
			↑	+
			?	O
			≤ 55 < others	O
			≤ 55: NR	O
			> 60 > others	O
			35–56 > others	
				O
	Comorbidity	Inflammatory arthritis	↑	O
		Oral anti-cancer agents	Charlson comorbidity index: ↑	O
			↓	
				O
	Comorbidity (physical)	Hepatitis C	↓	O
			?	O
		Chronic conditions	↓	O
		Cardiovascular conditions	↓	O
			↑	O
			↕	O
	Comorbidity (mental)	Parkinson disease	↓	O
		Hepatitis C	↓	+
			↕	O
			↑	O
			?	O
		Chronic conditions	↓	O
		Cardiovascular conditions	↓	+
			↕	O
			?	O
	Comorbidity (depression)	Oral anti-cancer agents	↓	+
		HIV	↓	+
		Cardiovascular conditions	↓	++
	Gender (female)	Chronic pain	↑	O
		Hepatitis C	↓	O
			↑	O
			?	O
		Inflammatory arthritis	↓	O
			↑	O
		Chronic conditions	↓	O
			?	O
		Oral anti-cancer agents	↓	O
			↑	O
			?	O
		Cardiovascular conditions	↑	+
			?	O
			↓	O
Health care system related	Co-payments	Inflammatory arthritis	↓	+
		Chronic conditions	↓	+
			↑	–
		Not restricted	↓	++
		Oral anti-cancer agents	Less than US$10 > more than US$10	O
			↑	O
			↓	O
		Cardiovascular conditions	↓	+
			No > yes	+
			Yes > no	O
			US$0 > US$1 to US$9	+
			US$0 > US$10 to US$29	+
	Medication costs	Inflammatory arthritis	↓	O
		Oral anti-cancer agents	↓	O
	Health insurance	Chronic conditions	↑	O
		Cardiovascular conditions	↑	O
			?	O

Effect direction. *↑* positive effect on adherence, *↓* negative effect on adherence, *↕* inconsistent effect direction, ? effect direction not or unclearly reported, *++* robust evidence for an impact, *+* some evidence for an impact, − probably no impact, O uncertain impact

#### Social and economic factors

The evidence for an impact of education on adherence was uncertain for most diseases/therapies. Some evidence for a positive impact of education on adherence was exclusively noted for cardiovascular conditions [23, 37]. The impact of employment was mostly uncertain. Some evidence for a positive impact was exclusively noted in HIV-infected patients [32, 34]. The other conditions that were investigated for this influencing factor (hepatitis C, inflammatory arthritis and cardiovascular conditions) showed inconsistent results and thus were judged as uncertain evidence [23, 27, 38]. For the analysis of the influence of ethnic status on adherence, we considered different comparisons because the grouping in primary studies differed widely. Some evidence exist for inflammatory arthritis and robust evidence for cardiovascular conditions (in the USA) that white ethnicity is associated with higher adherence [33, 38]. In HIV-infected patients, there was some evidence that white individuals are more adherent than black individuals [32]. The SRs of cardiovascular conditions showed some evidence that large ethnic groups are more adherent than ethnic minorities [37]. Among patients with chronic diseases and patients taking oral anticancer agents, there was some evidence that a better financial status has a positive influence on adherence [20, 39]. The impact of financial status was uncertain in Parkinson disease, hepatitis C and cardiovascular conditions [21, 23, 27, 36, 37]. The influence of the socioeconomic status was uncertain in inflammatory arthritis and patients taking oral anticancer agents [28, 38]. In cardiovascular conditions, some evidence exists that a higher socioeconomic status has a positive impact on adherence [29]. Marital status was investigated in the SRs on Parkinson disease, inflammatory arthritis, chronic diseases, HIV, patients taking oral anticancer agents and cardiovascular conditions. The results were very inconsistent, and consequently, the impact was judged as uncertain overall [20, 23, 32, 36, 38, 39]. In addition, the impact of social support was uncertain in all SRs [23, 28, 30, 37, 38].

#### Therapy-related factors

We found some evidence for a negative influence of intake of different medications in cardiovascular conditions. The impact of all other therapy related factors (duration of therapy, number of tablets, intake frequency, intake at meals) was uncertain in all conditions [23, 28, 35–39].

#### Disease-related factors

Duration of disease was the only disease-related factor considered in this overview. Most of the SRs that analysed this factor showed conflicting effect directions, and the evidence for an impact was thus judged as either uncertain or probably no impact overall [23, 27, 28, 35, 38, 39].

#### Patient-related factors

In six of eight conditions, positive effect directions for higher age were reported. In two conditions (cardiovascular conditions and Parkinson disease), some evidence of an impact was found, and the impact of the other four conditions/medications was uncertain [20, 23, 24, 28, 35–39]. In contrast, negative effect directions of higher age in chronic diseases, cardiovascular conditions and oral anticancer agents were reported [20, 21, 23, 24, 28, 39]. However, the evidence for an impact was uncertain. More distinct (no linear) age groups were compared in the SRs on adherence in inflammatory arthritis, chronic diseases, HIV-infected patients, patients taking oral anticancer agents and cardiovascular conditions [20, 21, 23, 28, 31, 32, 37–39]. In two conditions, there was some evidence for an impact. In HIV-infected patients, persons older than 45 years tend to be more adherent than those under 45 years [32]. In patients taking oral anticancer agents, there was some evidence that middle-aged people (approximately 45–60) are more adherent than very old (> 75 years) and younger people (< 45 years) [28]. General comorbidity or physical comorbidity was assessed in inflammatory arthritis [38], patients taking oral anticancer agents, hepatitis C, chronic diseases and cardiovascular conditions [20, 21, 27, 28, 37, 39]. Overall, positive as well as negative effect directions were reported in all included SRs, and the evidence was therefore judged to be uncertain. General mental comorbidity was considered a potential adherence-influencing factor in the conditions Parkinson disease, hepatitis C, chronic diseases and cardiovascular conditions. Negative effect directions were reported for most conditions, while the results were inconsistent in hepatitis C and cardiovascular conditions [20, 21, 27, 30, 36, 37]. The evidence for an impact was mostly judged as uncertain for this factor. Some evidence for a negative impact of mental comorbidity on medication adherence was exclusively noted in hepatitis C and cardiovascular conditions [21, 27, 30, 37]. Depression was analysed in patients taking oral anticancer agents, HIV infection or cardiovascular conditions. In patients taking oral anticancer agents and HIV-infected patients, some evidence was observed, and robust evidence for a negative impact was noted in cardiovascular conditions [28, 30, 32]. Gender was analysed in the SRs on chronic pain, hepatitis C, inflammatory arthritis, chronic diseases, oral anticancer agents and cardiovascular conditions [20, 21, 23, 27, 28, 33, 35, 37–39]. The impact was judged as uncertain in all SRs because the effect directions were conflicting (within and between SRs). Some evidence for higher adherence in women was noted exclusively in cardiovascular conditions [21, 23, 33, 37].

#### Health care system-related factors

For co-payments (any co-payment and higher co-payments), the effect direction was almost always negative. Some evidence for a negative impact of co-payments on adherence in inflammatory arthritis, chronic diseases and cardiovascular conditions exists [20, 22, 23, 25, 26, 38]. The meta-analysis of Sinnott et al. provides robust evidence for a negative impact of co-payments on adherence across different conditions [40]. The evidence for an impact was uncertain in oral-anticancer agents [39]. In cardiovascular conditions, there was some evidence that patients not paying any co-payments are more adherent than those patients paying (any) co-payments [25, 26]. Medication costs were analysed in patients with inflammatory arthritis and patients taking oral anticancer agents. Only negative effect directions were reported, but the evidence for a negative impact on adherence was uncertain in both conditions [38, 39]. It was uncertain whether health insurance status (insured vs. uninsured) influences adherence in patients with chronic or cardiovascular conditions [23, 25].

## Discussion

This overview includes 21 SRs on 313 individual primary studies in a broad spectrum of chronic conditions. Compared with the previous version, this focused update increases the certainty of evidence for some factors (e.g., co-payments or ethnic status) and identifies new evidence on other factors (socioeconomic status, depression and insurance status) [12].

We analysed seven potentially socioeconomic adherence-influencing factors. Although mostly positive effect directions were reported, the overall evidence for an impact is uncertain for employment and education. The evidence synthesis indicates that belonging to an ethnic minority seems to be associated with reduced adherence. In contrast, higher financial status and better socioeconomic position seem to have a positive impact on adherence. None of the therapy-related (but not therapy-specific) factors showed evidence for a strong impact on adherence. The same seems to be true for disease duration. Studies focusing on distinct age groups suggest that age does not have a linear association with adherence but that the association is rather a concave shape with an adherence peak in middle to older ages, i.e., adherence is particularly low in very young and very old persons. Studies that analysed age as a continuous linear variable and studies that dichotomized age showed inconsistent results. The explanation for the inconsistent results of the linear analyses might also be attributed to the fact that the association is indeed non-linear. Gender seems to have no consistent impact on adherence. Considering comorbidities, there was only robust evidence that depression impacts adherence negatively. We also found robust evidence that co-payments reduce adherence. Considering this information together with the socioeconomic factors and age suggests that there is a social gradient in adherence behaviour.

Although the majority of literature on adherence-influencing factors is overwhelming, we could only judge the influence for many factors as uncertain. In addition, from the high risk of bias, the main reason for so many uncertain judgements was imprecision. The main cause for downgrading due to imprecision was insufficient reporting, which prevented us from adequately assessing the results. For example, in many cases, we could not even use modified vote counting satisfactorily. Therefore, unclear impact ratings indicate that the evidence is insufficient to allow a conclusion not that there is the tendency that these factors have no impact.

Moreover, the results for many factors were inconsistent. Overviews of SRs are always at high risk for discordant or heterogeneous results across the included SRs [42]. We tried to prevent strong heterogeneity by focusing on factors for which we assumed homogeneity across different conditions and considering only implementation adherence to oral drugs. Nevertheless, the results of our overview were also partly heterogeneous. This is particularly true for the influencing factors education, employment, different medications, duration of disease and gender. One might argue that this suggests that the influence of these factors dependents on condition or setting. However, if inconsistency was observed, this was mostly true within as well as between SRs. Thus, we believe that positive findings might be caused by spurious findings in primary studies (confounding bias, type one error rate, selective reporting). A condition-related explanation for heterogeneity might be that many SRs seem to include symptomatic as well as asymptomatic patients. Research has shown that symptomatic patients are mostly more adherent than asymptomatic patients [43, 44]. This assumption is supported by the fact that especially therapy- and disease-related influencing factors, which are related to the symptomatic patients, were inconsistent. Moreover, none of the included SRs distinguishes intentional (conscious decision not to take medication) and unintentional adherence (forget to take medication); however, it strongly stands to reason that the influencing factors can depend on the underlying reasons for non-adherence [45]. Additional sources of inconsistency that we could not control for were different definitions and measurements of influencing factors (e.g., socioeconomic status) and even more adherence measures (e.g., self-reported vs. electronic monitoring, > 90% of pills taken vs. > 80% vs. mean intake).

We included SRs on any physical chronic diseases and analysed only factors we assumed were independent of disease/therapy. Therefore, on the one hand, we believe that our results are widely applicable for implementation adherence to oral drugs in physical chronic diseases. On the other hand, it should be considered in the interpretation of the findings that the influence of a factor might vary between region/setting. In particular, the influence of different ethnic groups probably depends on the country/region since an ethnic minority in one region could be an ethnic majority in another region However, although ethnic minorities are different ethnic groups in different countries, we believe that all ethnic minorities likely face similar adherence challenges independent of the country they live in.

The identified risk factors of non-adherence can indicate patients who are at increased risk for non-adherence. For clinical practice, this information can help identify and select patients who require support for being adherent. In studies on adherence, the information can help with the identification of relevant participants [46] or the development of adherence risk prediction models [47]. Moreover, the knowledge of influencing factors of adherence can support the development of tailored health technologies to increase adherence by treating the underlying barriers (e.g., depression treatment, reducing co-payments). In this regard, health policy decision makers should consider that there seems to be a social gradient in adherence.

Our overview has some methodological limitations. First, we limited our literature search to English and German languages because there were no other language skills in our research team and no resources for translating articles. Second, we used modified vote counting; however, we are aware that this type of methodology has strong limitations. Nevertheless, we decided to use modified vote counting because we anticipated that this is the only method to harmonize the results from different types of narrative synthesizes. Third, we only analysed therapy-unrelated factors. Consequently, regarding indications where therapy-related factors play an important role (e.g., adverse events in chemotherapy), our evidence is incomplete per se.

## Conclusion

There is sufficient evidence that depression and co-payments have a negative impact on adherence. Evidence suggests that general mental comorbidity and belonging to an ethnic minority might have a negative impact on adherence and that a higher socioeconomic status might have a positive impact on adherence. In addition, the evidence suggests that the influence of age on medication adherence has a concave pattern, i.e., lower adherence in young age groups, increasing adherence with a peak in middle to older age groups and lower adherence in very old age groups. The moderate to high risk of bias in the included SRs and the exclusion of 78 reviews due to missing quality assessment of included primary studies indicate that there is a need for more methodically sound research to provide stronger conclusions. Future primary studies and SRs should use validated adherence measures, adjust the analysis for relevant confounding factors, avoid using arbitrary cut-offs for influencing factors (e.g., age) and report the effect measures with 95% confidence intervals. Furthermore, the studies should analyse intentional and non-intentional adherence distinctly.

## Additional files

Additional file 1: Full search strategy. (DOCX 14 kb)

Additional file 2: List of excluded studies. (DOCX 29 kb)

Additional file 3: Cross Table. (XLSX 32 kb)

Additional file 4: Results of each individual included SR. (DOCX 19 kb)

## Abbreviations

CCA: Corrected covered area
GRADE: Grading of Recommendations, Assessment, Development and Evaluation
OoSRs: Overview of Systematic Reviews
PRISMA: Preferred Reporting Items for Systematic Reviews and Meta-Analyses
ROBIS: Risk of Bias in Systematic Reviews
SR: Systematic review
