# Supplementary material for: Medication adherence influencing factors—an (updated) overview of systematic reviews
Source: Syst Rev. 2019 May 10;8:112. doi: 10.1186/s13643-019-1014-8 (PMC6511120; doi:10.1186/s13643-019-1014-8)
Supplement: Supplementary file 1 — Full search strategy. (DOCX 14 kb) [file 13643_2019_1014_MOESM1_ESM.docx]

| **Complete search strategy EMBASE; Last updated: 13.06.2018** |
| --- |
| (adherence:ab,ti OR adherent:ab,ti OR adhere:ab,ti OR nonadherence:ab,ti OR nonadherent:ab,ti OR compliance:ab,ti OR ‘medication compliance’/exp OR compliant:ab,ti OR comply:ab,ti OR noncompliance:ab,ti OR noncompliant:ab,ti)  AND (‘risk factor’/exp OR factor:ab,ti OR factors:ab,ti OR predict:ab,ti OR predictor:ab,ti OR predictors:ab,ti OR indicate:ab,ti OR indicator:ab,ti OR indicators:ab,ti OR influence:ab,ti OR influencing:ab,ti OR determinate:ab,ti OR determinates:ab,ti OR determination:ab,ti OR barrier:ab,ti OR barriers:ab,ti OR facilitate:ab,ti OR facilitator:ab,ti OR facilitators:ab,ti OR hindrance:ab,ti OR hindrances:ab,ti OR age:ti,ab OR age factor/exp OR gender:ti,ab OR sex:ti,ab OR ‘gender identity”/exp OR sex/exp OR comorbidity/exp OR comorbidity:ab,ti OR ethnic groups/exp OR ethnic*:ti,ab OR education/exp OR education:ti,ab OR ‘employment status”/exp OR employ*:ti,ab OR unemploy*:ti,ab OR unemployment/exp OR income/exp OR income:ti,ab OR ‘financial situation”:ti,ab OR ‘financial status”:ti,ab OR ‘named groups by marital status”/exp OR marital:ti,ab OR married:ti,ab OR single:ti,ab OR ‘social support”/exp OR ‘social support”:ti,ab OR ‘regime complexity”:ti,ab OR (number AND (tablets:ti,ab OR medications:ti,ab OR pills:ti,ab OR day:ti,ab)) OR dose*:ti,ab OR mealtime*:ti,ab OR meal/exp OR meal:ti,ab OR ‘frequency of intake”:ti,ab OR ‘intake frequency”:ti,ab OR polypharmacy:ti,ab OR polypharmacy/exp OR ‘pill burden” OR (different:ti,ab OR various:ti,ab OR several:ti,ab AND (tablets:ti,ab OR medications:ti,ab OR medicaments:ti,ab OR prescriptions:ti,ab OR pills:ti,ab OR drugs:ti,ab)) OR (duration:ti,ab OR length:ti,ab AND (therapy:ti,ab OR treatment:ti,ab OR diseases:ti,ab illness:ti,ab)) OR ‘co-payment”:ti,ab OR ((medication:ti,ab OR drug:ti,ab) AND (costs:ti,ab)) OR ‘out of pocket costs”:ti,ab OR insurance:ti,ab OR insurance/exp)  AND (‘meta analysis”/mj OR 'systematic review'/mj OR (meta NEAR/1 analy*):ab,ti OR metaanalys*:ab,ti OR (systematic NEAR/1 review*):ab,ti OR ‘systematic literature review‘:ab,ti OR ((‘data extraction”:ab OR ‘selection criteria”:ab OR ‘inclusion criteria”:ab) AND review:ab,ti)) AND (english:la OR german:la) AND [embase]/lim AND ('article'/it OR 'article in press'/it OR 'review'/it) AND human/exp  AND [1-5-2014]/sd |
| **Complete seacrh strategy MEDLINE (via Pubmed); Last updated: 13.06.2018** |
| (Adherence [TIAB] OR adherent [TIAB] OR adhere [TIAB] OR nonadherence [TIAB] OR nonadherent [TIAB] OR Compliance [TIAB] OR “patient compliance” [MeSH Terms] OR medication adherence[mesh] OR compliant [TIAB] OR comply [TIAB] OR noncompliance [TIAB] OR noncompliant [TIAB])  AND (risk factors [mesh] OR factor [TIAB] OR factors [TIAB] OR predict [TIAB] OR predictor [TIAB] OR predictors [TIAB] OR indicate [TIAB] OR indicator [TIAB] OR indicators [TIAB] OR influence [TIAB] OR influencing [TIAB] OR determinate [TIAB] OR determinates [TIAB] OR determination [TIAB] OR barrier [TIAB] OR barriers [TIAB] OR facilitate [TIAB] OR facilitator [TIAB] OR facilitators [TIAB] OR age[tiab] OR age factor[mesh] OR gender[tiab] OR sex[tiab] OR gender identity[mesh] OR sex[mesh] OR comorbidity[tiab] OR comorbidity[mesh] OR ethnic groups[mesh] OR ethnic*[tiab] OR education[mesh] OR education[tiab] OR employment[mesh] OR employ*[tiab] OR unemploy*[tiab] OR unemployment[mesh] OR income[mesh] OR income[tiab] OR financial situation[tiab] OR financial status[tiab] OR marital status[mesh] OR marital[tiab] OR married[tiab] OR single[mesh] OR single[tiab] OR social support[mesh] OR social support[tiab] OR regime complexity [tiab] OR (number AND (tablets[tiab] OR medications[tiab] OR medicaments[tiab] OR prescriptions[tiab] OR pills[tiab] OR drugs[tiab])) OR dose*[tiab] OR mealtime*[tiab] OR meal[mesh] OR meal[tiab] OR “intake frequency”[tiab] OR polypharmacy[tiab] OR polypharmacy[mesh] OR “pill burden”[tiab] OR (different[tiab] OR various[tiab] OR several[tiab] AND (tablets[tiab] OR tablets[mesh] OR medications[tiab] OR pills[tiab])) OR (duration[tiab] OR length[tiab] AND (therapy[tiab] OR treatment[tiab] OR diseases[tiab] illness[tiab])) OR co payment[tiab] OR ((medication[tiab] OR drug[tiab]) AND (costs[tiab])) OR out of pocket costs[tiab] OR insurance[tiab] OR insurance[mesh])  AND ("Meta-Analysis" [Publication Type] OR "Meta-Analysis as Topic" [Mesh] OR “meta analysis” [TIAB] OR metaanalysis [TIAB] OR “systematic review”[TIAB] OR “systematic literature review” [TIAB] OR ((“selection criteria” [TIAB] OR “inclusion criteria”[TIAB] OR “data extraction”[TIAB]) AND (review [Publication Type] OR review[TIAB])))  NOT ("Comment" [Publication Type] OR "Letter" [Publication Type] OR "Editorial" [Publication Type]) AND (english [la] OR german [la]) AND human[mesh] |
