# Supplementary material for: Medication adherence influencing factors—an (updated) overview of systematic reviews
Source: Syst Rev. 2019 May 10;8:112. doi: 10.1186/s13643-019-1014-8 (PMC6511120; doi:10.1186/s13643-019-1014-8)
Supplement: Supplementary file 2 — List of excluded studies. (DOCX 29 kb) [file 13643_2019_1014_MOESM2_ESM.docx]

# List of excluded studies after full-text screening (with reasons)

| Reference | Main reason for exclusion |
| --- | --- |
| Abegaz, T.M., et al., Nonadherence to antihypertensive drugs: A systematic review and meta-analysis. Medicine (Baltimore), 2017. 96(4): p. e5641. | Study type: no SR according to our definition |
| Adams, A.S., S. Banerjee, and C.J. Ku, Medication adherence and racial differences in diabetes in the USA: An update. Diabetes Management, 2015. 5(2): p. 79-87. | Study type: no SR according to our definition |
| Adejumo, O.A., et al., Contemporary issues on the epidemiology and antiretroviral adherence of HIV-infected adolescents in sub-Saharan Africa: a narrative review. J Int AIDS Soc, 2015. 18: p. 20049. | Study type: no SR according to our definition |
| Ahmad, A. and K. Sorensen, Enabling and hindering factors influencing adherence to asthma treatment among adolescents: A systematic literature review. Journal of Asthma, 2016. 53(8): p. 862-878. | Study type: no SR according to our definition |
| Ammon, N., S. Mason, and J.M. Corkery, Factors impacting antiretroviral therapy adherence among human immunodeficiency virus-positive adolescents in Sub-Saharan Africa: a systematic review. Public Health, 2018. 157: p. 20-31. | Patients: no adult patients with physical chronic disease |
| Assawasuwannakit, P., R. Braund, and S.B. Duffull, A model-based meta-analysis of the influence of factors that impact adherence to medications. J Clin Pharm Ther, 2015. 40(1): p. 24-31. | Study type: no SR according to our definition |
| Banek, K., et al., Adherence to artemisinin-based combination therapy for the treatment of malaria: a systematic review of the evidence. Malar J, 2014. 13: p. 7. | Patients: no adult patients with physical chronic disease |
| Beinart, N.A., et al., Individual and intervention-related factors associated with adherence to home exercise in chronic low back pain: a systematic review. BMC Med Res Methodol, 2013. 13(134): p. 1471-2288. | Medication: not > 50 % oral intake |
| Belaiche, S., et al., Factors relevant to medication non-adherence in kidney transplant: a systematic review. Int J Clin Pharm, 2017. 39(3): p. 582-593. | Only statistically significant results reported |
| Bezabhe, W.M., et al., Adherence to Antiretroviral Therapy and Virologic Failure: A Meta-Analysis. Medicine (Baltimore), 2016. 95(15): p. e3361. | Study type: no SR according to our definition |
| Bolsewicz, K., et al., Factors associated with antiretroviral treatment uptake and adherence: a review. Perspectives from Australia, Canada, and the United Kingdom. AIDS Care, 2015. 27(12): p. 1429-38. | Study type: no SR according to our definition |
| Brundisini, F., et al., Type 2 diabetes patients' and providers' differing perspectives on medication nonadherence: a qualitative meta-synthesis. BMC Health Serv Res, 2015. 15: p. 516. | Study type: no SR according to our definition |
| Caldeira, D., A. Vaz-Carneiro, and J. Costa, The impact of dosing frequency on medication adherence in chronic cardiovascular disease: systematic review and meta-analysis. Rev Port Cardiol, 2014. 33(7-8): p. 431-7. | Study type: no SR according to our definition |
| Chia, L., E.A. Schlenk, and J. Dunbar-Jacob, Effect of personal and cultural beliefs on medication adherence in the elderly. Drugs and Aging, 2006. 23(3): p. 191-202. | Study type: no SR according to our definition |
| Chowdhury, R., et al., Adherence to cardiovascular therapy: A meta-analysis of prevalence and clinical consequences. European Heart Journal, 2013. 34(38): p. 2940-2948. | Study type: no SR according to our definition |
| Clay, P.G., et al., Meta-Analysis of Studies Comparing Single and Multi-Tablet Fixed Dose Combination HIV Treatment Regimens. Medicine (Baltimore), 2015. 94(42): p. e1677. | Exposure: does not address the predefined influencing factors |
| Claxton, A.J., J. Cramer, and C. Pierce, A systematic review of the associations between dose regimens and medication compliance. Clinical Therapeutics, 2001. 23(8): p. 1296-1310. | Study type: no SR according to our definition |
| Coleman, C.I., et al., Dosing frequency and medication adherence in chronic disease. Journal of Managed Care Pharmacy, 2012. 18(7): p. 527-539. | Study type: no SR according to our definition |
| Conn, V.S., et al., Interventions to improve medication adherence among older adults: Meta-analysis of adherence outcomes among randomized controlled trials. Gerontologist, 2009. 49(4): p. 447-462. | Exposure: does not address the predefined influencing factors |
| Conn, V.S., et al., Cultural relevance in medication adherence interventions with underrepresented adults: systematic review and meta-analysis of outcomes. Prev Med, 2014. 69: p. 239-47. | Exposure: does not address the predefined influencing factors |
| Cox, L. and J. Hunt, Factors that affect adolescents' adherence to diabetes treatment. Nurs Child Young People, 2015. 27(1): p. 16-21. | Patients: no adult patients with physical chronic disease |
| Cramer, J.A., A Systematic Review of Adherence with Medications for Diabetes. Diabetes Care, 2004. 27(5): p. 1218-1224. | Study type: no SR according to our definition |
| Croome, N., et al., Patient-reported barriers and facilitators to antiretroviral adherence in sub-Saharan Africa. Aids, 2017. 31(7): p. 995-1007. | Study type: no SR according to our definition |
| Czarny, M.J., et al., Adherence to dual antiplatelet therapy after coronary stenting: a systematic review. Clin Cardiol, 2014. 37(8): p. 505-13. | Study type: no SR according to our definition |
| De Vera, M.A., et al., Medication adherence in gout: a systematic review. Arthritis Care Res (Hoboken), 2014. 66(10): p. 1551-9. | Study type: no SR according to our definition |
| DiMatteo, M.R., Social Support and Patient Adherence to Medical Treatment: A Meta-Analysis. Health Psychology, 2004. 23(2): p. 207-218. | Study type: no SR according to our definition |
| DiMatteo, M.R., Evidence-based strategies to foster adherence and improve patient outcomes. Epilepsy Behav, 2004. 5(6): p. 1017-20. | Study type: no SR according to our definition |
| Detsis, M., et al., Factors Associated with HIV Testing and HIV Treatment Adherence: A Systematic Review. Curr Pharm Des, 2017. 23(18): p. 2568-2578. | Study type: no SR according to our definition |
| Falagas, M.E., et al., Socioeconomic status (SES) as a determinant of adherence to treatment in HIV infected patients: A systematic review of the literature. Retrovirology, 2008. 5((Falagas M.E., m.falagas@aibs.gr; Zarkadoulia E.A., e.zarkadoulia@aibs.gr; Pliatsika P.A., e.pliatsika@aibs.gr) Alfa Institute of Biomedical Sciences (AIBS), Athens, Greece). | Study type: no SR according to our definition |
| Fidder, H.H., et al., Low rates of adherence for tumor necrosis factor-alpha inhibitors in Crohn's disease and rheumatoid arthritis: results of a systematic review. J Adv Nurs, 2014. 70(3): p. 476-96. | Exposure: does not address the predefined influencing factors |
| Foot, H., et al., The necessity-concerns framework predicts adherence to medication in multiple illness conditions: A meta-analysis. Patient Educ Couns, 2016. 99(5): p. 706-717. | Study type: no SR according to our definition |
| Fu, L., Y. Hu, and H.Z. Lu, Overviews of reviews on patient compliance with medication protocols used in highly active antiretroviral therapy. International Journal of Nursing Sciences, 2015. 2(1): p. 61-65. | Study type: no SR according to our definition |
| Gathe, J.C., Improving patient adherence with antiretroviral therapy: evaluation of once-daily administration of didanosine. Ann Pharmacother, 2004. 38(7-8): p. 1236-42. | Study type: no SR according to our definition |
| Gajria, K., et al., Adherence, persistence, and medication discontinuation in patients with attention-deficit/hyperactivity disorder - A systematic literature review. Neuropsychiatric Disease and Treatment, 2014. 10((Gajria K.; Sikirica V., vsikirica@shire.com) Global Health Economics, Outcomes Research and Epidemiology, Shire, Wayne, PA, United States): p. 1543-1569. | Study type: no SR according to our definition |
| Garcia-Llana, H., et al., The role of depression, anxiety, stress and adherence to treatment in dialysis patients health-related quality of life: a systematic review of the literature. Nefrologia, 2014. 34(5): p. 637-57. | Study type: no SR according to our definition |
| Ghimire, S., et al., Nonadherence to Medication Therapy in Haemodialysis Patients: A Systematic Review. PLoS One, 2015. 10(12): p. e0144119. | Only significant results reported |
| Goldman, D.P., G.F. Joyce, and Y. Zheng, Prescription drug cost sharing: associations with medication and medical utilization and spending and health. Curr Med Res Opin, 2007. 23(8): p. 1903-12. | Study type: no SR according to our definition |
| Gorczynski, P., et al., Are people with schizophrenia adherent to diabetes medication? A comparative meta-analysis. Psychiatry Res, 2017. 250: p. 17-24. | Study type: no SR according to our definition |
| Grenard, J.L., et al., Depression and medication adherence in the treatment of chronic diseases in the United States: A meta-analysis. Journal of General Internal Medicine, 2011. 26(10): p. 1175-1182. | Study type: no SR according to our definition |
| Griva, K., et al., Non-adherence in patients on peritoneal dialysis: a systematic review. PLoS One, 2014. 9(2): p. e89001. | Medication: not > 50 % oral intake |
| Gupta, A.K., S. Arshad, and N.R. Poulter, Compliance, safety, and effectiveness of fixed-dose combinations of antihypertensive agents: A meta-analysis. Hypertension, 2010. 55(2): p. 399-407. | Study type: no SR according to our definition |
| Happe, L.E., et al., A systematic literature review assessing the directional impact of managed care formulary restrictions on medication adherence, clinical outcomes, economic outcomes, and health care resource utilization. J Manag Care Spec Pharm, 2014. 20(7): p. 677-84. | Study type: no SR according to our definition |
| Heyer, A. and G.A. Ogunbanjo, Adherence to HIV antiretroviral therapy. Part I: A review of factors that influence adherence. South African Family Practice, 2006. 48(8): p. 5-9. | Study type: no SR according to our definition |
| Holmes, E.A., D.A. Hughes, and V.L. Morrison, Predicting adherence to medications using health psychology theories: a systematic review of 20 years of empirical research. Value Health, 2014. 17(8): p. 863-76. | Exposure: does not address the predefined influencing factors |
| Hu, L., et al., Nonadherence to the medical regimen after lung transplantation: A systematic review. Heart Lung, 2017. 46(3): p. 178-186. | Study type: no SR according to our definition |
| Hucker, A., et al., Non-adherence to immunosuppressants following renal transplantation: a protocol for a systematic review. BMJ Open, 2017. 7(9): p. e015411. | Only protocol available |
| Hudelson, C. and L. Cluver, Factors associated with adherence to antiretroviral therapy among adolescents living with HIV/AIDS in low- and middle-income countries: a systematic review. AIDS Care, 2015. 27(7): p. 805-16. | Patients: no adult patients with physical chronic disease |
| Hutchins, V., et al., A systematic review of adherence, treatment satisfaction and costs, in fixed-dose combination regimens in type 2 diabetes. Current Medical Research and Opinion, 2011. 27(6): p. 1157-1168. | Study type: no SR according to our definition |
| Iskedjian, M., et al., Relationship between daily dose frequency and adherence to antihypertensive pharmacotherapy: Evidence from a meta-analysis. Clinical Therapeutics, 2002. 24(2): p. 302-316. | Study type: no SR according to our definition |
| Johnston, N., et al., Systematic reviews: Causes of non-adherence to P2Y12 inhibitors in acute coronary syndromes and response to intervention. Open Heart, 2016. 3(2). | Study type: no SR according to our definition |
| Kane, S.V., Systematic review: Adherence issues in the treatment of ulcerative colitis. Alimentary Pharmacology and Therapeutics, 2006. 23(5): p. 577-585. | Study type: no SR according to our definition |
| Keller, D.L., J. Wright, and H.A. Pace, Impact of health literacy on health outcomes in ambulatory care patients: A systematic review. Annals of Pharmacotherapy, 2008. 42(9): p. 1272-1281. | Exposure: does not address the predefined influencing factors |
| Krass, I., P. Schieback, and T. Dhippayom, Adherence to diabetes medication: a systematic review. Diabet Med, 2015. 32(6): p. 725-37. | Study type: no SR according to our definition |
| Kothawala, P., et al., Systematic review and meta-analysis of real-world adherence to drug therapy for osteoporosis. Mayo Clinic Proceedings, 2007. 82(12): p. 1493-1501. | Study type: no SR according to our definition |
| Kruk, M.E. and N. Schwalbe, The relation between intermittent dosing and adherence: Preliminary insights. Clinical Therapeutics, 2006. 28(12): p. 1989-1995. | Study type: no SR according to our definition |
| Kubica, A., et al., Adherence to antiplatelet treatment with P2Y12 receptor inhibitors. Is there anything we can do to improve it? A systematic review of randomized trials. Curr Med Res Opin, 2016. 32(8): p. 1441-1451. | Study type: no SR according to our definition |
| Ladova, K., et al., Healthy adherer effect - the pitfall in the interpretation of the effect of medication adherence on health outcomes. BMC Health Serv Res, 2013. 13(461): p. 1472-6963. | Study type: no SR according to our definition |
| Lakanpaul, et al., A systematic review of explanatory factors of barriers and facilitators to improving asthma management in South Asian children. Cancer Epidemiol. 2014 Jun;38(3):214-226. doi: 10.1016/j.canep.2014.03.012. Epub 2014 Apr 24. | Exposure: does not address the predefined influencing factors |
| Lee, W.C., et al., Prevalence and economic consequences of medication adherence in diabetes: A systematic literature review. Managed Care Interface, 2006. 19(7): p. 31-41. | Study type: no SR according to our definition |
| Lemstra, M. and M.W. Alsabbagh, Proportion and risk indicators of nonadherence to antihypertensive therapy: A meta-analysis. Patient Preference and Adherence, 2014. 8((Lemstra M., mark.lemstra@usask.ca; Alsabbagh M.W.) Academic Family Medicine, College of Medicine, University of Saskatchewan, Saskatoon, SK, Canada): p. 211-218. | Study type: no SR according to our definition |
| Lennon, O., et al., Lifestyle interventions for secondary disease prevention in stroke and transient ischaemic attack: A systematic review. European Journal of Preventive Cardiology, 2014. 21(8): p. 1026-1039. | Study type: no SR according to our definition |
| Li, W., Z.M. Zhang, and X.L. Jiang, Once daily vs multiple daily mesalamine therapy for mild to moderate ulcerative colitis: a meta-analysis. Colorectal Disease, 2016. 18(7): p. O214-O223. | Study type: no SR according to our definition |
| Lin, S. and G.J. Melendez-Torres, Systematic review of risk factors for nonadherence to TB treatment in immigrant populations. Trans R Soc Trop Med Hyg, 2016. 110(5): p. 268-280. | Outcome adherence |
| Lin, C., et al., Breast cancer oral anti-cancer medication adherence: a systematic review of psychosocial motivators and barriers. Breast Cancer Res Treat, 2017. 165(2): p. 247-260. | Study type: no SR according to our definition |
| Loke, Y.K., et al., Systematic review of consistency between adherence to cardiovascular or diabetes medication and health literacy in older adults. Annals of Pharmacotherapy, 2012. 46(6): p. 863-872. | Exposure: does not address the predefined influencing factors |
| Lopez-Gonzalez, R., et al., Adherence to biologic therapies and associated factors in rheumatoid arthritis, spondyloarthritis and psoriatic arthritis: a systematic literature review. Clin Exp Rheumatol, 2015. 33(4): p. 559-69. | Medication: not > 50 % oral intake |
| Low, J.K., et al., Interventions to improve medication adherence in adult kidney transplant recipients: a systematic review. Nephrol Dial Transplant, 2015. 30(5): p. 752-61. | Exposure: does not address the predefined influencing factors |
| Lutge, E.E., et al., Incentives and enablers to improve adherence in tuberculosis. Cochrane Database Syst Rev, 2015(9): p. CD007952. | Exposure: does not address the predefined influencing factors |
| MacPherson, P., et al., Service delivery interventions to improve adolescents' linkage, retention and adherence to antiretroviral therapy and HIV care. Trop Med Int Health, 2015. 20(8): p. 1015-32. | Exposure: does not address the predefined influencing factors |
| Makanjuola, T., H.B. Taddese, and A. Booth, Factors associated with adherence to treatment with isoniazid for the prevention of tuberculosis amongst people living with HIV/AIDS: a systematic review of qualitative data. PLoS One, 2014. 9(2): p. e87166. | Exposure: does not address the predefined influencing factors |
| Malta, M., et al., Adherence to antiretroviral therapy for human immunodeficiency virus/acquired immune deficiency syndrome among drug users: A systematic review. Addiction, 2008. 103(8): p. 1242-1257. | Study type: no SR according to our definition |
| Mamudu, H.M., et al., The effects of coronary artery calcium screening on behavioral modification, risk perception, and medication adherence among asymptomatic adults: a systematic review. Atherosclerosis, 2014. 236(2): p. 338-50. | Study type: no SR according to our definition |
| Marshall, I.J., C.D.A. Wolfe, and C. McKevitt, Lay perspectives on hypertension and drug adherence: Systematic review of qualitative research. BMJ (Online), 2012. 345(7867). | Exposure: does not address the predefined influencing factors |
| Mausbach, B.T., R.B. Schwab, and S.A. Irwin, Depression as a predictor of adherence to adjuvant endocrine therapy (AET) in women with breast cancer: a systematic review and meta-analysis. Breast Cancer Res Treat, 2015. 152(2): p. 239-46. | Study type: no SR according to our definition |
| McCullough, A., et al., Interventions for enhancing adherence to treatment in adults with bronchiectasis. Cochrane Database Syst Rev, 2015(11): p. CD011023. | Exposure: does not address the predefined influencing factors |
| McCullough, A.R., et al., Behavior change theory, content and delivery of interventions to enhance adherence in chronic respiratory disease: A systematic review. Respiratory Medicine, 2016. 116((McCullough A.R., amccullo@bond.edu.au; Macindoe C.; Yii N.) Centre for Research in Evidence-based Practice, Faculty of Health Sciences and Medicine, Bond University, Robina, Australia): p. 78-84. | Exposure: does not address the predefined influencing factors |
| McGrady, M.E. and K.A. Hommel, Medication adherence and health care utilization in pediatric chronic illness: A systematic review. Pediatrics, 2013. 132(4): p. 730-740. | Patients: no adult patients with physical chronic disease |
| McKenzie, S.J., et al., The Burden of Non-Adherence to Cardiovascular Medications Among the Aging Population in Australia: A Meta-Analysis. Drugs and Aging, 2015. 32(3): p. 217-225. | Exposure: does not address the predefined influencing factors |
| McSharry, J., et al., Perceptions and experiences of taking oral medications for the treatment of Type 2 diabetes mellitus: a systematic review and meta-synthesis of qualitative studies. Diabetic Medicine, 2016. 33(10): p. 1330-1338. | Study type: no SR according to our definition |
| Mhaskar, R., et al., Adherence to antiretroviral therapy in India: a systematic review and meta-analysis. BMC Fam Pract, 2013. 14(105): p. 1471-2296. | Study type: no SR according to our definition |
| Mikyas, Y., I. Agodoa, and N. Yurgin, A systematic review of osteoporosis medication adherence and osteoporosis-related fracture costs in men. Appl Health Econ Health Policy, 2014. 12(3): p. 267-77. | Exposure: does not address the predefined influencing factors |
| Milazi, M., A. Bonner, and C. Douglas, The effectiveness of education or behavioral interventions on adherence to phosphate control in adults receiving hemodialysis: a systematic review protocol. JBI Database System Rev Implement Rep, 2015. 13(3): p. 91-102. | Exposure: does not address the predefined influencing factors |
| Mogre, V., et al., Adherence to self-care behaviours and associated barriers in type 2 diabetes patients of low-and middle-income countries: a systematic review protocol. Syst Rev, 2017. 6(1): p. 39. | Only protocol available |
| Morrissey, E.C., et al., Effectiveness and content analysis of interventions to enhance medication adherence in hypertension: a systematic review and meta-analysis protocol. Syst Rev, 2016. 5: p. 96. | Exposure: does not address the predefined influencing factors |
| Mountain, E., et al., Antiretroviral therapy uptake, attrition, adherence and outcomes among HIV-infected female sex workers: a systematic review and meta-analysis. PLoS One, 2014. 9(9): p. e105645. | Exposure: does not address the predefined influencing factors |
| Nachega, J.B., et al., Community-Based Interventions to Improve and Sustain Antiretroviral Therapy Adherence, Retention in HIV Care and Clinical Outcomes in Low- and Middle-Income Countries for Achieving the UNAIDS 90-90-90 Targets. Curr HIV/AIDS Rep, 2016. 13(5): p. 241-255. | Exposure: does not address the predefined influencing factors |
| Nachega, J.B., et al., Lower pill burden and once-daily antiretroviral treatment regimens for HIV infection: A meta-analysis of randomized controlled trials. Clin Infect Dis, 2014. 58(9): p. 1297-307. | Exposure: does not address the predefined influencing factors |
| Nachega, J.B., et al., Adherence to antiretroviral therapy during and after pregnancy in low-income, middle-income, and high-income countries: A systematic review and meta-analysis. AIDS, 2012. 26(16): p. 2039-2052. | Study type: no SR according to our definition |
| Naderi, S.H., J.P. Bestwick, and D.S. Wald, Adherence to drugs that prevent cardiovascular disease: Meta-analysis on 376,162 patients. American Journal of Medicine, 2012. 125(9): p. 882-887. | Study type: no SR according to our definition |
| Nagata, J.M., L.R. Gatti, and F.K. Barg, Social determinants of iron supplementation among women of reproductive age: A systematic review of qualitative data. Maternal and Child Nutrition, 2012. 8(1): p. 1-18. | Study type: no SR according to our definition |
| Newman-Casey, P.A., M. Dayno, and A.L. Robin, Systematic review of educational interventions to improve glaucoma medication adherence: An update in 2015. Expert Review of Ophthalmology, 2016. 11(1): p. 5-20. | Exposure: does not address the predefined influencing factors |
| Nielsen, J., et al., Non-adherence to anti-hypertensive medication in low- and middle-income countries: A systematic review and meta-analysis of 92443 subjects. Journal of Human Hypertension, 2017. 31(1): p. 14-21. | Study type: no SR according to our definition |
| Nieuwlaat, R., et al., Interventions for enhancing medication adherence. Cochrane Database Syst Rev, 2014(11): p. CD000011. | Exposure: does not address the predefined influencing factors |
| Ortego, C., et al., Sex differences in adherence to highly active antiretroviral therapy: a meta-analysis. J Am Med Inform Assoc, 2012. 19(5): p. 696-704. | Study type: no SR according to our definition |
| Parienti, J.J., et al., Better adherence with once-daily antiretroviral regimens: A meta-analysis. Clinical Infectious Diseases, 2009. 48(4): p. 484-488. | Study type: no SR according to our definition |
| Patton, D.E., et al., Theory-Based Interventions to Improve Medication Adherence in Older Adults Prescribed Polypharmacy: A Systematic Review. Drugs and Aging, 2016((Patton D.E.; Hughes C.M.; Cadogan C.A.; Ryan C.A., cristinryan@rcsi.ie) School of Pharmacy, Queen’s University Belfast, Belfast, United Kingdom): p. 1-17. | Exposure: does not address the predefined influencing factors |
| Peltzer, K. and S. Pengpid, Socioeconomic factors in adherence to HIV therapy in low- and middle-income countries. Herz, 2013. 38(6): p. 578-86. | Study type: no SR according to our definition |
| Puts M. T. E. , et al., Factors influencing adherence to cancer treatment in older adults with cancer: a systematic review. Annals of Oncology 2014. 25(3): p. 564-577 | Medication: not > 50 % oral intake |
| Rash, J.A., et al., A systematic review of interventions to improve adherence to statin medication: What do we know about what works? Prev Med, 2016. 90((Rash J.A.; Campbell T.S., t.s.campbell@ucalgary.ca) Department of Psychology, University of Calgary, Calgary, Canada): p. 155-169. | Exposure: does not address the predefined influencing factors |
| Reardon, G., S. Kotak, and G.F. Schwartz, Objective assessment of compliance and persistence among patients treated for glaucoma and ocular hypertension: A systematic review. Patient Preference and Adherence, 2011. 5((Reardon G., greardon@informagenics.com) Informagenics, LLC, Worthington, OH, United States): p. 441-463. | Study type: no SR according to our definition |
| Rollason, V. and N. Vogt, Reduction of polypharmacy in the elderly: A systematic review of the role of the pharmacist. Drugs and Aging, 2003. 20(11): p. 817-832. | Exposure: does not address the predefined influencing factors |
| Ruppar, T.M., et al., Medication adherence interventions improve heart failure mortality and readmission rates: Systematic review and meta-analysis of controlled trials. Journal of the American Heart Association, 2016. 5(6). | Exposure: does not address the predefined influencing factors |
| Ruppar, T.M., et al., Systematic Review of Clinical Practice Guidelines for the Improvement of Medication Adherence. Int J Behav Med, 2015. 22(6): p. 699-708. | Exposure: does not address the predefined influencing factors |
| Rybacki, J.J., Improving cardiovascular health in postmenopausal women by addressing medication adherence issues. J Am Pharm Assoc (Wash), 2002. 42(1): p. 63-71; quiz 72-3. | Study type: no SR according to our definition |
| Saini, S.D., et al., Effect of medication dosing frequency on adherence in chronic diseases. J Behav Med, 2009. 32(5): p. 406-28. | Study type: no SR according to our definition |
| Salema, N.E., R.A. Elliott, and C. Glazebrook, A systematic review of adherence-enhancing interventions in adolescents taking long-term medicines. Nestle Nutr Workshop Ser Pediatr Program, 2011. 68: p. 49-61. | Exposure: does not address the predefined influencing factors |
| Sandelowski, M., et al., A systematic review comparing antiretroviral adherence descriptive and intervention studies conducted in the USA. AIDS Care - Psychological and Socio-Medical Aspects of AIDS/HIV, 2009. 21(8): p. 953-966. | Study type: no SR according to our definition |
| Santer, M., et al., Treatment non-adherence in pediatric long-term medical conditions: Systematic review and synthesis of qualitative studies of caregivers' views. BMC Pediatrics, 2014. 14(1). | Study type: no SR according to our definition |
| Santo, K., et al., Interventions to improve medication adherence in coronary disease patients: A systematic review and meta-analysis of randomised controlled trials. European Journal of Preventive Cardiology, 2016. 23(10): p. 1065-1076. | Exposure: does not address the predefined influencing factors |
| Sapkota, S., et al., A systematic review of interventions addressing adherence to anti-diabetic medications in patients with type 2 diabetes--impact on adherence. PLoS One, 2015. 10(2): p. e0118296. | Exposure: does not address the predefined influencing factors |
| Sattler, E.L., J.S. Lee, and M. Perri, 3rd, Medication (re)fill adherence measures derived from pharmacy claims data in older Americans: a review of the literature. PLoS Med, 2013. 10(3): p. 12. | Exposure: does not address the predefined influencing factors |
| Scheiman-Elazary, A., et al., The Rate of Adherence to Antiarthritis Medications and Associated Factors among Patients with Rheumatoid Arthritis: A Systematic Literature Review and Metaanalysis. J Rheumatol, 2016. 43(3): p. 512-23. | Study type: no SR according to our definition |
| Schroeder, K., T. Fahey, and S. Ebrahim, How Can We Improve Adherence to Blood Pressure-Lowering Medication in Ambulatory Care? Systematic Review of Randomized Controlled Trials. Archives of Internal Medicine, 2004. 164(7): p. 722-732. | Exposure: does not address the predefined influencing factors |
| Schroeder, K., T. Fahey, and S. Ebrahim, Interventions for improving adherence to treatment in patients with high blood pressure in ambulatory settings. Diabetes Care, 2004. 27(5): p. 1218-24. | Exposure: does not address the predefined influencing factors |
| Sherrill, B., et al., Single-Pill vs Free-Equivalent Combination Therapies for Hypertension: A Meta-Analysis of Health Care Costs and Adherence. Journal of Clinical Hypertension, 2011. 13(12): p. 898-909. | Exposure: does not address the predefined influencing factors |
| Shubber, Z., et al., Patient-Reported Barriers to Adherence to Antiretroviral Therapy: A Systematic Review and Meta-Analysis. PLoS Med, 2016. 13(11). | Study type: no SR according to our definition |
| Simoens, S. and P.R. Sinnaeve, Patient co-payment and adherence to statins: A review and case studies. Cardiovascular Drugs and Therapy, 2014. 28(1): p. 99-109. | Study type: no SR according to our definition |
| Smith, D., et al., A systematic review of medication non-adherence in persons with dementia or cognitive impairment. PLoS One, 2017. 12(2): p. e0170651. | Study type: no SR according to our definition |
| Snyder, S., et al., Medical adherence to acne therapy: A systematic review. American Journal of Clinical Dermatology, 2014. 15(2): p. 87-94. | Study type: no SR according to our definition |
| Soboka, M. and G.T. Feyissa, The effectiveness of counseling, material support and/or nutritional supplementation on improving adherence to anti-retroviral therapy and clinical outcomes among HIV patients: A systematic review of quantitative evidence protocol. JBI Database System Rev Implement Rep, 2015. 13(7): p. 142-152. | Study type: no SR according to our definition |
| Taylor, G.H., S.L. Wilson, and J. Sharp, Medical, psychological, and sociodemographic factors associated with adherence to cardiac rehabilitation programs: a systematic review. Ann Pharmacother, 2010. 44(12): p. 1968-75. | Study type: no SR according to our definition |
| Thorneloe, R.J., et al., Adherence to medication in patients with psoriasis: A systematic literature review. British Journal of Dermatology, 2013. 168(1): p. 20-31. | Study type: no SR according to our definition |
| Treuer, T., et al., Factors affecting treatment adherence to atomoxetine in ADHD: A systematic review. Neuropsychiatric Disease and Treatment, 2016. 12((Treuer T., treuer_tamas@lilly.com) Neuroscience Research, Eli Lilly and Company, Budapest, Hungary): p. 1061-1083. | Study type: no SR according to our definition |
| Uthman, O.A., et al., Depression and adherence to antiretroviral therapy in low-, middle- and high-income countries: A systematic review and meta-analysis. Curr HIV/AIDS Rep, 2014. 11(3): p. 291-307. | Study type: no SR according to our definition |
| van der Laan, D.M., et al., Factors associated with antihypertensive medication non-adherence: a systematic review. J Hum Hypertens, 2017. 31(11): p. 687-694. | Study type: no SR according to our definition |
| van Driel, M.L., et al., Interventions to improve adherence to lipid-lowering medication. Cochrane Database of Systematic Reviews, 2016. 2016(12). | Exposure: does not address the predefined influencing factors |
| Viswanathan, M., et al., Interventions to improve adherence to self-administered medications for chronic diseases in the United States: a systematic review. Helicobacter, 2012. 17(5): p. 374-81. | Exposure: does not address the predefined influencing factors |
| Walsh, K.E., et al., Medication adherence among pediatric patients with sickle cell disease: a systematic review. Pediatrics, 2014. 134(6): p. 1175-83. | Patients: no adult patients with physical chronic disease |
| Weaver, M.S., et al., Interventions to improve adherence to treatment for paediatric tuberculosis in low- and middle-income countries: A systematic review and meta-analysis. Bull World Health Organ, 2015. 93(10): p. 700-711B. | Patients: no adult patients with physical chronic disease |
| Xu, A., T. Chomutare, and S. Iyengar, Persuasive attributes of medication adherence interventions for older adults: a systematic review. Technol Health Care, 2014. 22(2): p. 189-98. | Exposure: does not address the predefined influencing factors |
| Yap, A.F., T. Thirumoorthy, and Y.H. Kwan, Systematic review of the barriers affecting medication adherence in older adults. Geriatrics and Gerontology International, 2016. 16(10): p. 1093-1101. | Study type: no SR according to our definition |
| Zeber, J.E., et al., A systematic literature review of psychosocial and behavioral factors associated with initial medication adherence: A report of the ISPOR medication adherence & persistence special interest group. Value in Health, 2013. 16(5): p. 891-900. | Study type: no SR according to our definition |
| Zwikker, H.E., et al., Psychosocial predictors of non-adherence to chronic medication: Systematic review of longitudinal studies. Patient Preference and Adherence, 2014. 8((Zwikker H.E.; van den Bemt B.J., b.vandenbemt@maartenskliniek.nl; Vriezekolk J.E.; van den Ende C.H.) Departments of Rheumatology and Pharmacy, Netherlands): p. 519-563. | Exposure: does not address the predefined influencing factors |
