# Supplementary material for: Medication adherence influencing factors—an (updated) overview of systematic reviews
Source: Syst Rev. 2019 May 10;8:112. doi: 10.1186/s13643-019-1014-8 (PMC6511120; doi:10.1186/s13643-019-1014-8)
Supplement: Supplementary file 4 — Results of each individual included SR. (DOCX 19 kb) [file 13643_2019_1014_MOESM4_ESM.docx]

| ***Study*** | **Results explorative (effect direction/compared categories; comparisons showing effect direction; significant comparisons showing effect direction; total number of comparisons/heterogeneity; risk of bias)** |
| --- | --- |
| **Aziz 2016** | Age: ↑; 4; NR; 5; Moderate  Age: ↓; 1; NR; 5; Low  Age 65 and older > younger than 65: 1; NR; 1; Low  Gender: male > female; 3; NR; 4; Moderate  Gender: NR; NR; NR, 4; Moderate  No comorbidities (physical) > comorbidities (physical): 3; NR; 3; Moderate  No comorbidities (mental) > comorbidities (mental): 3; NR; 3; Low  Health Insurance > no health insurance: 2; NR; 2; Low  Income: ↑; 5; NR; 5; Low  Co-payment: ↓; 12; NR; 13; Moderate  Co-payment: ↑: 1; NR; 13; Low  Marital status: NR; 1; NR; 1; Moderate |
| **Broekmans 2008** | Age: ↑; NR; 2; NR; Unclear  Duration of disease: NR; NR; 0; 1; Unclear  Education: NR; NR; 0; 2; High  Gender: Women > men; NR; 1; 2; Moderate  Different medications: ↓ ;1; 1; 1; Unclear  Different medications: ↑; 1;1; 1; Moderate |
| **Chen 2015** | Age: ≤ 55 < others: 1; 1; 3; Low  Age: ≤ 55: NR; NR; 0; 3; Low  Age: ↓; 1; 1; 3; Low  Gender: female > male: 1; 1; 2; Low  Gender: NR; NR; 0; 2; Low  Ethnic status: White > Non-White: 1; 1; 1; Low  Ethnic status: Non-Asian > Asian: 1; 1; 1; Low  Income: NR; 1; 0; 1; Low  Comorbidities (physical): ↓; 3; 3; 3; Low  Comorbidities (mental): ↓; 2; 2; 3; Low  Comorbidities (mental): NR; 1; 0; 3 Low |
| **Crawshaw 2016** | No comorbidities (mental) > comorbidities (mental): 9; 5; 9; Moderate  Social support: ↑; 3; 2; 3; Moderate |
| **Daley 2012** | Age: ↑; 2; 2; 2; Moderate  Comorbidity (mental): ↓; NR; 3; NR; High  Different medications: ↓; NR; 2; NR; Moderate  Education: ↑; NR; 2; NR; Moderate  Frequency of intake: ↑; NR; 1; NR; Moderate  Income: ↑; 1; 0; 1; Moderate  Marital status / social support: ↑; 1; 0; 1; Moderate |
| **Gourzoulidis 2017** | Co-payment: ↓; 16; 11; 16; High |
| **Jaam 2017** | Age: ↑; 2; 2; 13; Low  Age: ↓; 2; 2; 13; High  Age: NR; NR; 2; NR; High  Age: > 60 > others; 1; 1; 1; High  Gender: female > male 3; 3; 17; Moderate  Gender: male > female; 2; 2; 17; High  Gender: NR; 12; 0; 17; High  Marital status: ↑; 3; 3; 9; High  Marital status: NR; 6; 0; 9; Moderate  Education: ↑; 8; 8; 20; High  Education: ↓; 1; 1; 20; Moderate  Education: NR; 11; 0; NR; High  Employed: ↑; 2; 2; 10; Moderate  Employed: ↓; 1; 1; 10; Low  Employed: NR; 7; 0; 10; Moderate  Income: ↑; 5; 5; 6; High  Income: NR; 1; 0; 6; Moderate  Insurance: ↑; 1; 1; 2; Moderate  Insurance: NR; 1; 0; 2; Moderate  Duration of disease: ↑; 1; 1; 13; Low  Duration of disease: ↓; 2; 2; 13; High  Duration of disease: NR; 10; 0; 10; Moderate  Number of medications: ↓; 7; 7; 9; High  Number of medications: NR; 2; 0; 9; High  Co-payment: ↓; 2; 2; 2; Moderate  Social support: ↑; 1; 1; 1; Low |
| **Krueger 2015** | Age: ↑; 7; 7; 17 Moderate Age: NR; 10; 0; 17; High |
| **Maimaris 2013** | Health insurance: insured > uninsured; 2; 0; 3; Moderate  Health insurance: NR; 1; NR; 3; Moderate  Co-payment: US$0 > US$1 to US$9; 1; 1; 1; Moderate  Co-payment: US$0 > US$10 to US$29; 1; 1; 1; Moderate  Co-payment: ↓; 2; 2; 2; High  Co-payment: no co-payments > co-payments; 3; 3; 4; High  Co-payments: co-payments > no co-payments; 1; 1; 4; Low |
| **Mann 2014** | No co-payments > co-payments: 3; 3; 3, Moderate |
| **Mathes 2014(a)** | Age: NR; 4; 1; 5; High  Age: < 55 > others; 1; 1; 5; High  Gender: male > female; 2; 2; 6; High  Gender: female > male; 1; 0; 6; High  Gender: NR; 3; 0; 6; High  Ethnic status: NR; 1; 0; 2; High  Ethnic status: others > African American; 1; 0; 2; High  No comorbidities (physical) > comorbidities (physical): 1; 0; 2; Moderate  Comorbidities (physical): NR; 1; 0; 2; Moderate  No comorbidities (mental) > comorbidities (mental); 5; 3; 8; High  No comorbidities (mental) = comorbidities (mental): 1; 0; 8; Moderate  No comorbidities (mental) < comorbidities (mental): 1; NR; 8; High  No comorbidities (mental): NR; 1; 0; 8; Moderate  Duration of Disease: ↑; 1; 0; 1; Moderate  Education: high > low; 1; 0; 2; Moderate  Education: College > others; 1; 0; 2; High  Employment status: unemployed > employed; 2; 0; 2; High  No relationship > in a relationship; 1; 0; 1; High |
| **Mathes 2014(b)** | Age: middle age > very old (≥75) > young (≤45): 3; 3; 3; Moderate  Age: middle age (41 - 60) > others; 1; 0; 1; High  Age: ↑; 5; 3;10; High  Age; NR; 3; 0; 10; High  Age; ↓; 2; 0; 10; High  Ethnic status: NR; 3; 0; 3; High  Ethnic status: white > black; 1; 1; 1; Moderate  Ethnic status: white > asian; 1; 0; 1; Moderate  Ethnic status: white > hispanic; 1; 0; 1; Moderate  Ethnic status: white > non-white; 1; 1; 1; Moderate  Gender: NR; 2; 0; 5; High  Gender: female > male; 1; NR; 5; High  Gender: male > female; 2; 1; 5; Moderate  Marital status: married > unmarried; 1; 1; 2; Moderate  Marital status: NR; 1; 0; 2; High  Education: NR; 1; 0; 2; High  Education: secondary > primary; 1; NR; 1; High  Education: university > primary; 1; NR; 1; High  Education: primary > others; 1; NR; 1; High  Education: ↑; 1; NR; 2; High  Education: middle school or higher > less than middle school; 1; 0; 1; Moderate  Socioeconomic status: NR; 1; 0; 1; Moderate  Socioeconomic status ↑: 1; 1; 1; High  Socioeconomic status: higher quintiles > lowest quintile: 1; 0; 1; Moderate  Social support: NR; 1; 0; 3; High  Social support: ↑; 1; 1; 3; High  Social support > no social support: 1; 1; 3; Moderate  Duration of disease: NR; 1; 0; 1; High  Duration of disease: ↓; 1; NR; 1; High  Duration of disease: ↑; 2; 1; 2; High  Comorbidities: Charlson comorbidity index: ↓; 1; 1; 3; ModerateComorbidities: Charlson comorbidity index: ↑; 2; 2; 3; Moderate  Comorbidities (depression): yes vs no; 3; 1; 3; High  Duration of therapy: NR; 4; 1; 4; High  Duration of therapy: 1 year > 3 or 5 years; 1; NR; 1; High  Duration of therapy: more than 2 years > 0-2 years; 1; 0; 1; Moderate  Duration of therapy: ↓; 2; NR; 2; High  Number of tablets: NR; 1; 0; 2; High  Number of tablets: 2 > 1; 1; NR; 2; High  Number of medications: ↑; 2; NR; 4; High  Number of medications: ↓; 1; 1; 4; Low  Number of medications: NR; 1; 0; 4; Moderate  Co-payment: ↑; 1; 0; 2; High  Co-payment: less than 10 $ > more than 10 $; 1; 1; 2; Moderate |
| **Oosterom-Calo 2013** | Age: ↑; NR; 3; 7; Moderate  Age: 35-56 > others; 1; 1; 1; Moderate  Comorbidities (mental): ↓; 2; 2; 3; Moderate  Comorbidities (mental): ↕; 1; 1; 3; Low  Comorbidities (physical): ↑; NR; 3; 7; Moderate  Comorbidities (physical): ↓; NR; 2; 7; Moderate  Comorbidities (physical): ↕; 1; 1; 7; Moderate  Different medications: NR; NR; 1; 2; High  Education: NR; NR; NR; 2; Moderate  Ethnic status: ethnic minorities (e.g. African Americans) < majority ethnic groups (e.g. Caucasians); NR; 3; 5; Moderate  Financial status: NR; NR; 0; 1; Moderate  Frequency of intake: NR; NR; 0; 1; Moderate  Gender: Men > women: NR; 3; 7; Moderate  Gender: Women > men: NR; 2; 7; Moderate  Number of pills taken per day: NR; NR; 1; 2; Moderate  Social support: ↕; 2; 2; 2; Moderate |
| **Pasma 2013** | Age: ↑; NR; 3; 10; High  Age: 55-64 > others; 1; 1; 10; Moderate  Comorbidity: ↑; NR; 2; 5; Moderate  Co-payments: ↓; 1; 1; 1; Low  Different medications: ↑; NR; 1; 3; Moderate  Duration of disease: ↓; NR; 1; 9; High  Employed fulltime: NR; 3; 3; 3; High  Ethnic status: white > others; 1; 1; 1; Moderate  Frequency of intake: NR; NR; 0; 2; High  Gender: Men > Women: NR; 1; 10; Moderate  Gender: Women > men: NR; 1; 10; Moderate  Medication costs: ↓; 1; 1; 1; Low  Single / divorced: ↓; NR; 1; 4; High  Social support: ↑; NR; 1; 4; High  Socioeconomic status: ↓; NR; 1; 4; High |
| **Verbrugghe 2012** | Age: <45 < others; 1; 1; NR; Moderate  Age: ≤45 or ≥85 vs. others; 1; 1; NR; High  Age: ↑; 1; 1; NR; High  Age: ↓; 2; 2; 2; Moderate  Comorbidity: ↓; 2; 2; NR; Moderate  Co-payments: ↓; 2; 2; NR; Moderate  Different medications: ↓; 2; 2; NR; Moderate  Duration of disease: ↓; 1; 1; NR; Moderate  Duration of therapy: ↓; 3; 3; NR; High  Education: ↑; 1; 1; NR; Moderate  Ethnic status: African American > others; 1; 1; NR; Moderate  Ethnic status: non-white > others; 1; 1; NR; Moderate  Gender: men > women; 1; 1; NR; Moderate  Gender: women > men; 1; 1; NR; Moderate  Income: ↑; 1; 1; NR; High  Living alone < others; 1; 1; NR; Moderate  Marital status: non-married > married; 1; 1; NR; Moderate  Medication costs: ↓; 1; 1; NR; Moderate  Taking medication not at meal times; ↓; 1; 1; NR; Moderate |

NR: not applicable; NR: not reported

| ***Study*** | **Results focused (effect direction or compared categories; relative effect size; 95%-CI; number of studies; number of patients; heterogeneity; risk of bias)** |
| --- | --- |
| **Alsabbagh 2014** | Low Socioeconomic status vs. high socioeconomic status; RR = 0.89; 0.87 to 0.92; K = 40; n = 1371953; I² = 95%; Moderate |
| **Crawshaw 2016** | No comorbidity (depression) vs. comorbidity (depression); OR = 2.00; 1.57 to 3.33; K = 7; n = 5058; I² = 62%; Moderate |
| **Ghidei 2013** | Age < 45 years vs. > 45 years; RR = 0.72; 0.64 to 0.82; K = 12; n = 11325; I² = 37%; Moderate |
| **Hiko 2012** | Age 18-40 vs. Age > 41 years; OR = 0.53; 0.15 to 1.93; K = 2; n = 435; I² = 73%; Low  White vs. black; OR = 1.38; 1.21 to 1.58; K = 2; n = 7883; I² = 0%; Low  Employed vs. Unemployed; OR = 1.49; 0.74 to 3.01; K = 4; n = 1367; I² = 84%; Low  Low education vs. high education; OR = 0.99; 0.37 to 2.66; K = 4; n = 1183; I² = 52%; Low  Married vs. Divorced/widowed/single; OR = 0.84; 0.60 to 1.18; K = 2; n = 782; I² = 0%; Low  No comorbidity (depression) vs. comorbidity (depression); OR = 1.77; 1.17 to 2.69; K = 2; n = 653; I² = 0%; Low |
| **Lewey 2013** | Male vs. female; OR = 1.10; 1.07 to 1.13; K = 51; n = 2663638; I² = 95%; Low  White vs. non-white; OR = 1.53; 1.25 to 1.87; K = 11; n = 1027648; I² = 98%; Moderate |
| **Nachega 2015** | Employed vs. unemployed; OR = 1.27; 1.04 to 1.55; K = 28; n = 8743; I² = 77%; Moderate |
| **Sinnott 2013** | No co-payments vs. co-payments; OR = 1.11; 1.09 to 1.14; K = 7; n = 199996; I^2^ = 7 %; High |
